# Novel Full-Length Dissection of the Sympathetic Chain and Spinal Cord From T1-L1 in a Human Cadaver

**DOI:** 10.7759/cureus.102671

**Published:** 2026-01-30

**Authors:** Carolyn Enochs, Alice Li, Quinn Fan, Sara Richmond, Erin Paton, George Prada

**Affiliations:** 1 Department of Clinical Anatomy, Sam Houston State University College of Osteopathic Medicine, Conroe, USA

**Keywords:** dorsal root ganglia, dorsal rootlets, dura mater, gray rami, spinal cord, sympathetic chain, sympathetic ganglion, t1-l2, ventral rootlets, white rami

## Abstract

Anatomical investigations of the sympathetic chain have traditionally relied on regionally limited or anterior evisceration-based dissections, restricting appreciation of its longitudinal organization and relationships to the spinal cord. To date, no human cadaveric study has demonstrated a continuous bilateral in situ dissection of the paravertebral sympathetic chain from T1 to L1 in continuity with the spinal cord. This technical report describes a novel, reproducible posterior cadaveric dissection that preserves the bilateral sympathetic trunks, paravertebral ganglia, interganglionic fibers, rami communicantes, and native vertebral relationships. A systematic posterior approach involving removal of posterior musculature, ribs, costovertebral and costotransverse joints, and controlled reduction of vertebral bodies enabled exposure of the spinal cord from the brainstem to the cauda equina while maintaining adjacent neural integrity. This uninterrupted preparation permits direct visualization of segmental and longitudinal sympathetic connectivity and offers a previously undescribed anatomical perspective of the thoracolumbar sympathetic system. The technique provides substantial educational value and has important implications for surgical planning and anatomical research.

## Introduction

The sympathetic chain (also termed the sympathetic trunk or paravertebral ganglia) is a paired longitudinal structure that is a principal component of the sympathetic division of the autonomic nervous system (ANS). It consists of a series of interconnected ganglia running bilaterally alongside the vertebral column from the base of the skull to the coccyx. The cell bodies of presynaptic neurons of the sympathetic division of the ANS originate in the intermediolateral gray matter of the thoracic (T1-T12) and upper lumbar (L1-L2, occasionally L3) spinal cord segments [1]. This system regulates essential physiological functions, including pupillary dilation, as well as cardiovascular, respiratory, gastrointestinal, genitourinary, and nociceptive pathways [2]. Despite its broad clinical relevance, a fully comprehensive anatomical characterization of the human sympathetic chain remains absent from the current literature [3].

Previous investigations have primarily examined isolated regions of the sympathetic chain or have relied on animal models rather than intact human specimens. For example, Le Corre et al. characterized thoracolumbar spinal cord injury using a nonhuman primate model [4]. Anatomical studies by Govender et al., Civelek et al., and Saylam et al. focused exclusively on the cervical and upper thoracic portions of the sympathetic chain [3,5,6]. Similarly, Street et al., Chung et al., Zhang et al., Kommuru et al., and Peetermans et al. examined the upper thoracic chain or individual rami communicantes using anterior evisceration-based dissection approaches [7-11]. Although these studies quantified ganglia, documented anatomical variations, and identified key neural structures, they were limited to short thoracic segments (typically T1-T5) and required removal of thoracic viscera, precluding preservation of the sympathetic chain within its intact vertebral and spinal context.

To address this significant anatomical gap, we present the first complete posterior cadaveric dissection of the entire thoracolumbar sympathetic chain and spinal cord, preserving bilateral sympathetic trunks, all ganglia from T1 to L2, interganglionic fibers, white and gray rami communicantes, and vertebral-level relationships in situ. This uninterrupted preparation demonstrates the full course of the thoracolumbar sympathetic outflow in human anatomy and provides a comprehensive structural foundation for future investigations in autonomic neuroanatomy, neurophysiology, surgical planning, and disease modeling.

## Technical report

Vertebral spinous process exposure

**Figure 1:**
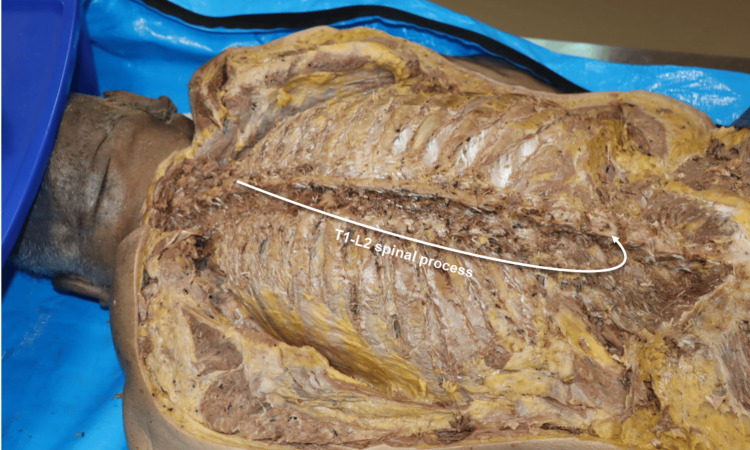
Exposure of the cadaveric vertebral spinous processes from T1 through L2. The donor was positioned prone (face down). From the reader’s perspective, the head is oriented toward the left side of the image. The spinous processes from T1 through T12 and extending inferiorly to L1–L2 are identified.

A systematic dissection was performed using a surgical scalpel, Metzenbaum and iris curved scissors, mosquito clamps, and DeBakey and Adson forceps to remove the skin, subcutaneous adipose tissue, and posterior musculature. This exposure allowed visualization of the thoracic and lumbar vertebral spinous processes and lamina from T1 to L2 in a 75-year-old male embalmed formalin-fixed donor (Figure 1).

Laminectomy

**Figure 2:**
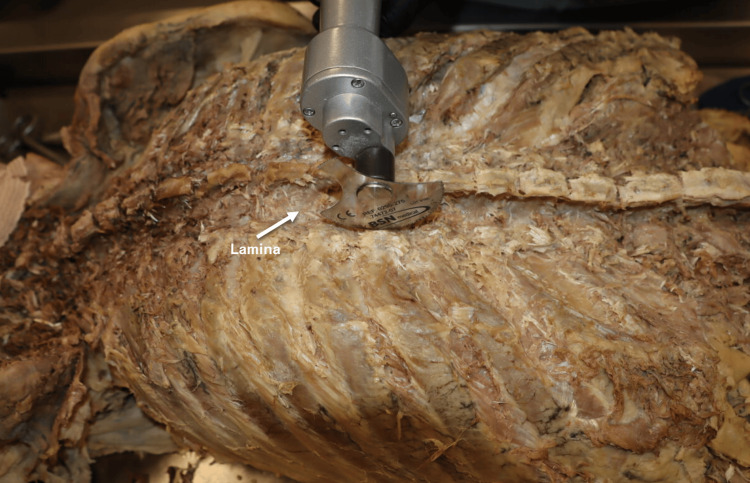
Laminectomy Procedure. Horizontal posterior view of the donor positioned prone (face down). From the reader’s perspective, the head (not visualized in this image) is oriented to the left.

After exposure of the vertebral laminae, a laminectomy was performed using an American Orthopaedic cast cutter saw (BSN Medical Inc., Charlotte, NC, USA) fitted with a Mopec autopsy blade (Figure 2). Bilateral vertical cuts were created through the laminae, and each laminar segment was removed sequentially by gentle elevation of the corresponding spinous process. The superior inner surface of the spinal canal was subsequently smoothed with a 1/2-inch chisel to minimize the risk of inadvertent injury to the underlying spinal cord.

Spinal cord and spinal nerves

**Figure 3:**
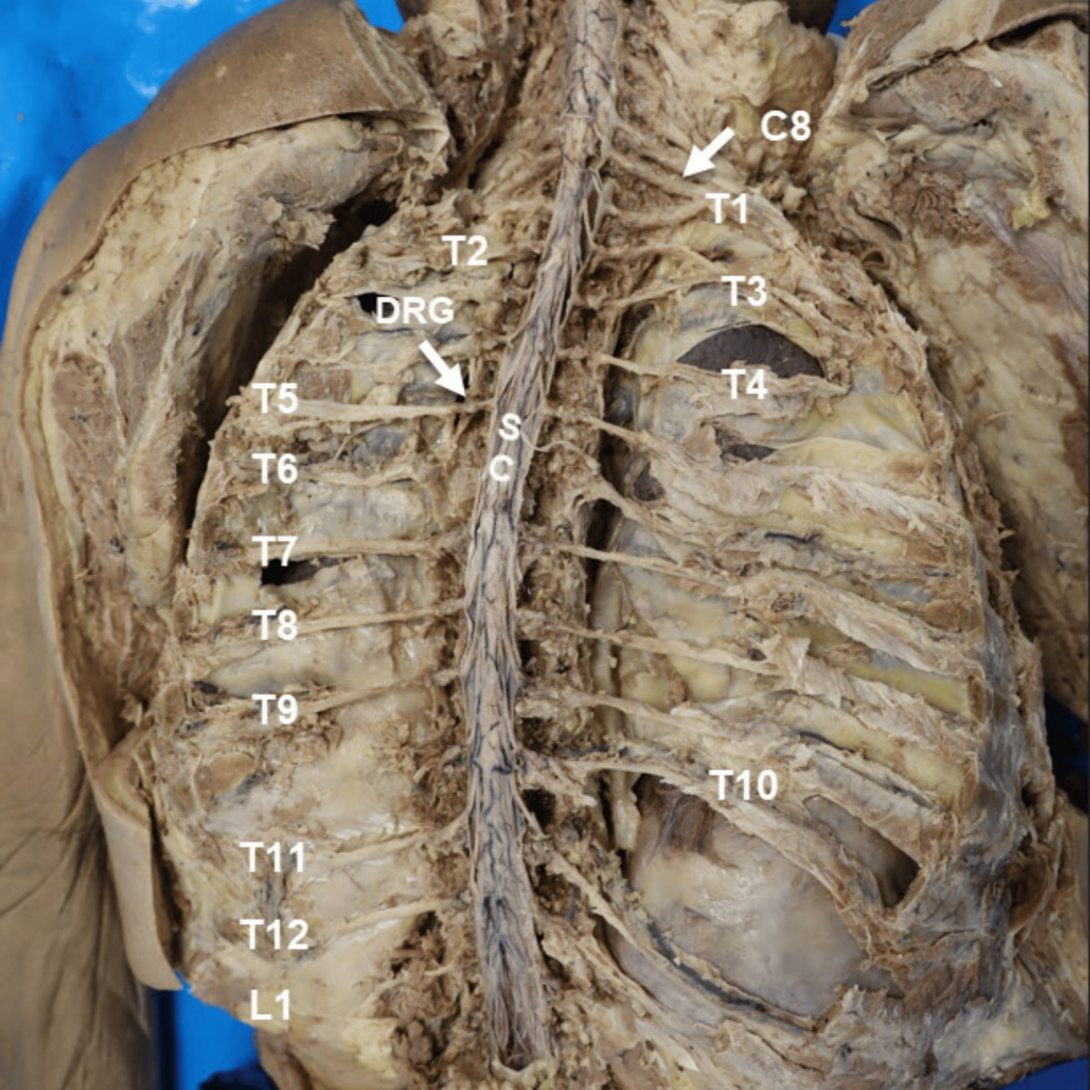
Spinal Cord and Spinal Nerves. Posterior vertical view of the donor in the prone (face-down) position. The head, not visible in this image, is oriented superior to the level of the C8 spinal nerve. From the reader’s perspective, the left and right sides of the image correspond to the donor’s anatomical left and right, respectively. Segmental visualization of the thoracolumbar spinal nerves (T1–L1) is demonstrated. The dura mater was longitudinally incised to expose the spinal cord, permitting clear identification of the spinal nerve roots. The dorsal root ganglion (DRG) at T5 and the spinal cord (SC) are labeled.

The spinal cord was exposed following a multilevel laminectomy performed from the upper thoracic to upper lumbar regions. After removal of the posterior elements, the dura mater was longitudinally transected along its midline to fully reveal the spinal cord, including the origins and trajectory of the segmental spinal roots and the thoracolumbar spinal nerves from T1 to L1 (Figure 3). To improve visualization of the lateral nerve pathways, the ribs were carefully detached and trimmed along their posterior arcs, allowing access to the intervertebral foramina and the emerging spinal nerve segments. The costovertebral and costotransverse joints were then meticulously disconnected using 1/2- to 1-inch chisels, which permitted complete exposure of the dorsal root ganglia and their anatomical relationships to the thoracic vertebrae and surrounding structures.

Brain and spinal cord

**Figure 4:**
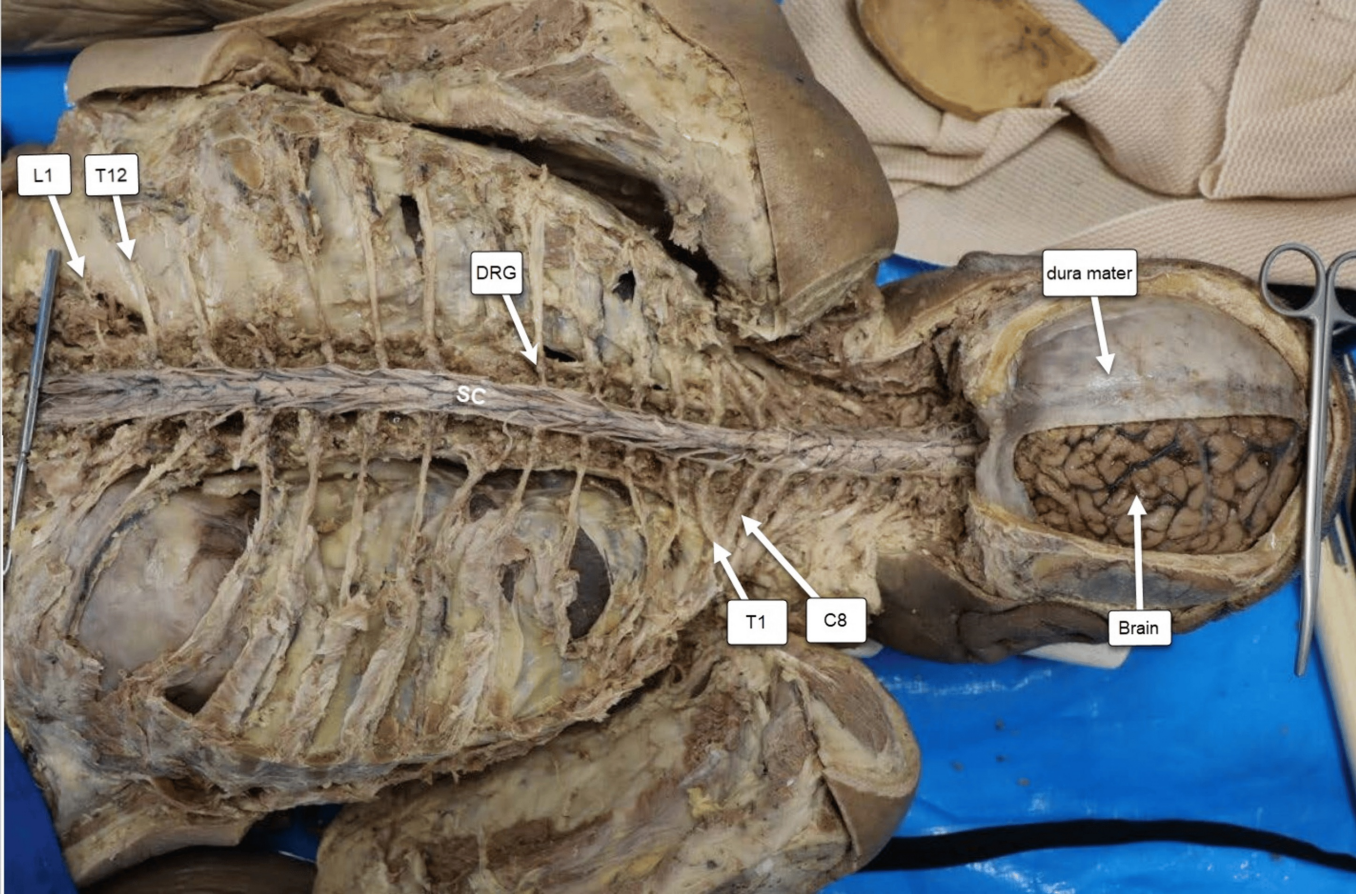
Brain and Spinal Cord View. In this view, the donor is positioned prone (face down). From the reader’s perspective, the donor’s left upper limb is oriented superiorly and the right upper limb inferiorly. C8 = eighth cervical spinal nerve; T1 = first thoracic spinal nerve; T12 = 12th thoracic spinal nerve; L1 = first lumbar spinal nerve; DRG = dorsal root ganglion.

The spinal cord was dissected in its entirety from the cervical segment (C1) through the lumbar segment (L1). The brain remained partially attached to the dura mater and fully continuous with the brainstem, which in turn remained adherent to the spinal cord (Figure 4). In this preparation, each spinal nerve from T1 to L1, along with its corresponding dorsal root ganglion (DRG), can be clearly visualized and traced from superior to inferior.

Left paravertebral sympathetic chain and dorsal root ganglia

**Figure 5:**
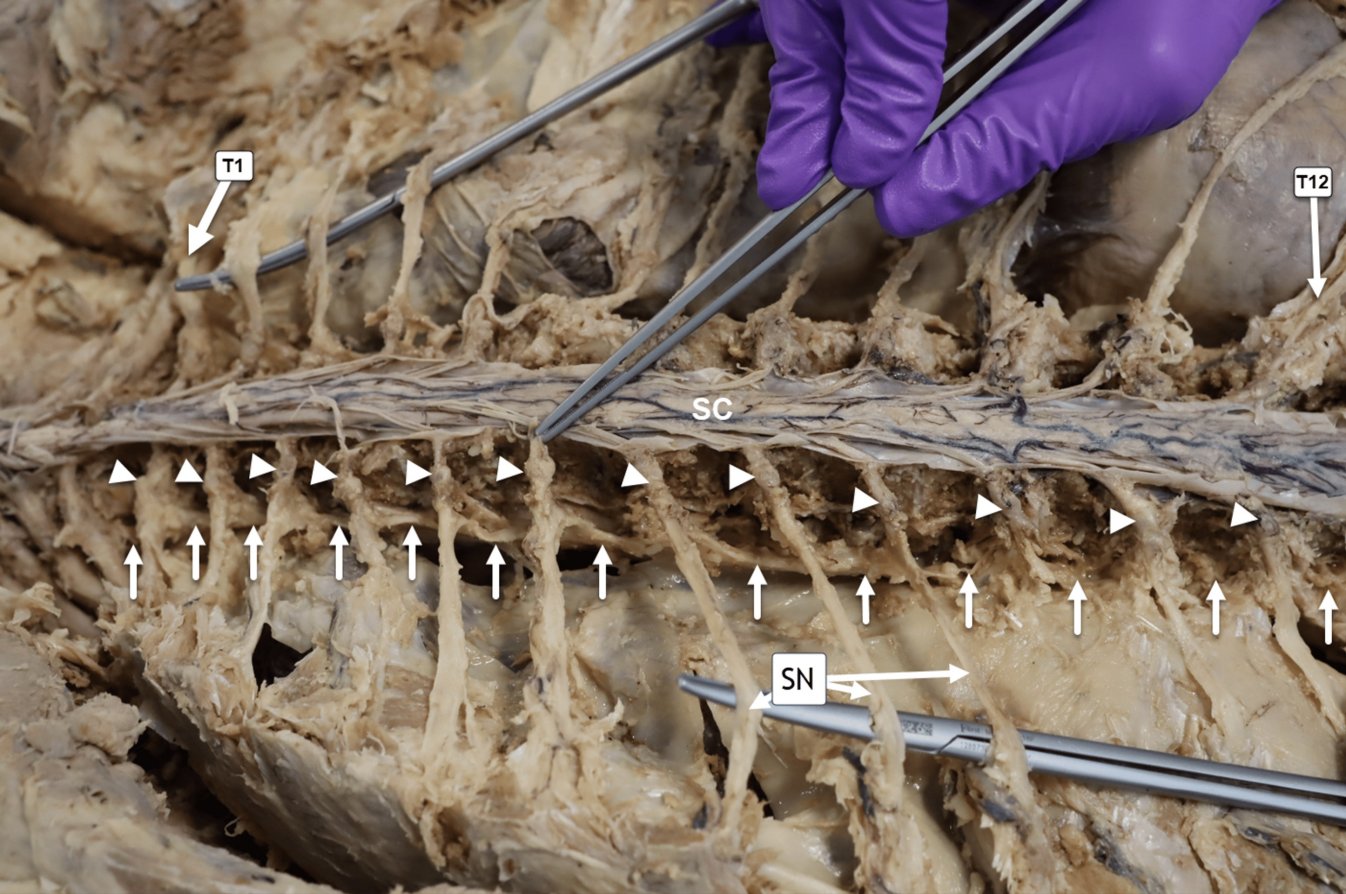
Posterior Approach of the Left Sympathetic Chain. Horizontal posterior view of the donor in the prone (face-down) position. From the reader’s perspective, the donor’s head, not visible in this image, is oriented laterally toward the left side of the image. The left paravertebral sympathetic chain from T1 to T12 is displayed. White arrowheads indicate the dorsal root ganglia (DRG), and solid white arrows trace the course of the sympathetic chain. Three thoracic spinal nerves (SN) are visible. SC = spinal cord.

The vertebral bodies (VB) were reduced to approximately half their original size using 1/2- and 1/4-inch chisels, carefully scraping each VB. The costovertebral and costotransverse joints, along with the ribs, were removed to expose the paravertebral sympathetic chain and the dorsal root ganglia in this specimen (Figure 5). Surrounding vasculature, including arteries and veins, was transected.

Right paravertebral sympathetic chain and dorsal root ganglia

**Figure 6:**
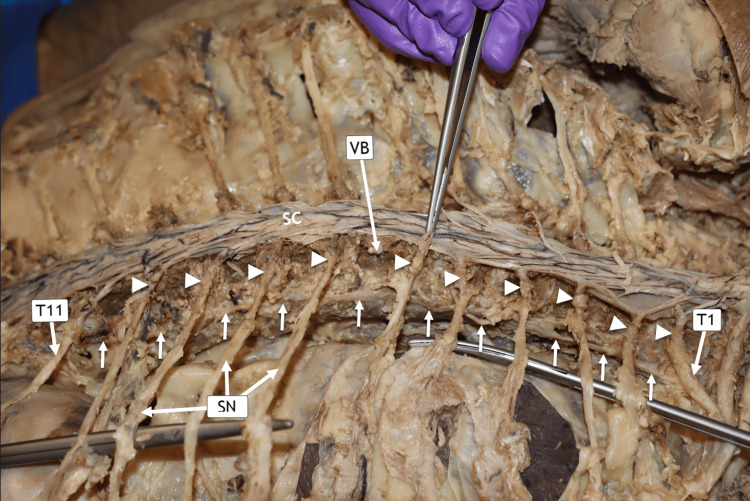
Posterior Approach of the Right Sympathetic Chain. Horizontal posterior view of the donor in the prone (face-down) position. From the reader’s perspective, the donor’s head, not visible in this image, is oriented laterally toward the right side of the image. The right paravertebral sympathetic chain is indicated by solid arrows, and the corresponding dorsal root ganglia (DRG) from T1 to T11 are denoted by arrowheads. The spinal nerves (SN) at T7–T9 are clearly visualized. VB = vertebral body

In this donor, the right PSC was located along the inferior half of the VB margins, a finding that may be related to the scoliosis present in this cadaver. The sympathetic chain is indicated by solid arrows, and its corresponding dorsal root ganglia from T1 to T11 are shown superior to it (Figure 6).

Left paravertebral sympathetic chain ganglia

**Figure 7:**
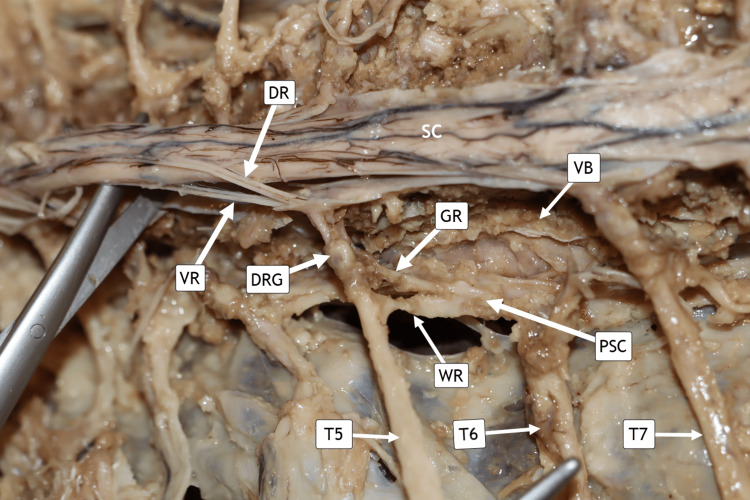
Left Side Paravertebral Sympathetic Chain. Horizontal posterior view of the donor in the prone position (face-down). The head, not visible, is oriented laterally toward the left. The paravertebral sympathetic chain (PSC) is shown between T5 and T7. Dorsal (DR) and ventral root fibers (VR) of T6 were transected to expose the vertebral bodies (VB) and clarify segmental anatomy. Dorsal root ganglia (DRG), grey rami (GR), and white rami (WR) are indicated. T5–T7 = thoracic spinal nerves five through seven.

Following the complete novel dissection, the left PSC at the level of the T5 segmental spinal nerve is demonstrated (Figure 7). The sensory dorsal rootlets (DR) are shown entering the spinal cord, while the motor ventral rootlets (VR) are shown exiting it. The cell bodies of the sensory neurons reside within the DRG, and the T5 spinal nerve contains both DR and VR fibers. The gray and white rami communicantes are clearly exposed after the VB was carefully reduced to half of the original size using chisels in this specimen. 

Spinal cord rootlets T10 to L1

**Figure 8:**
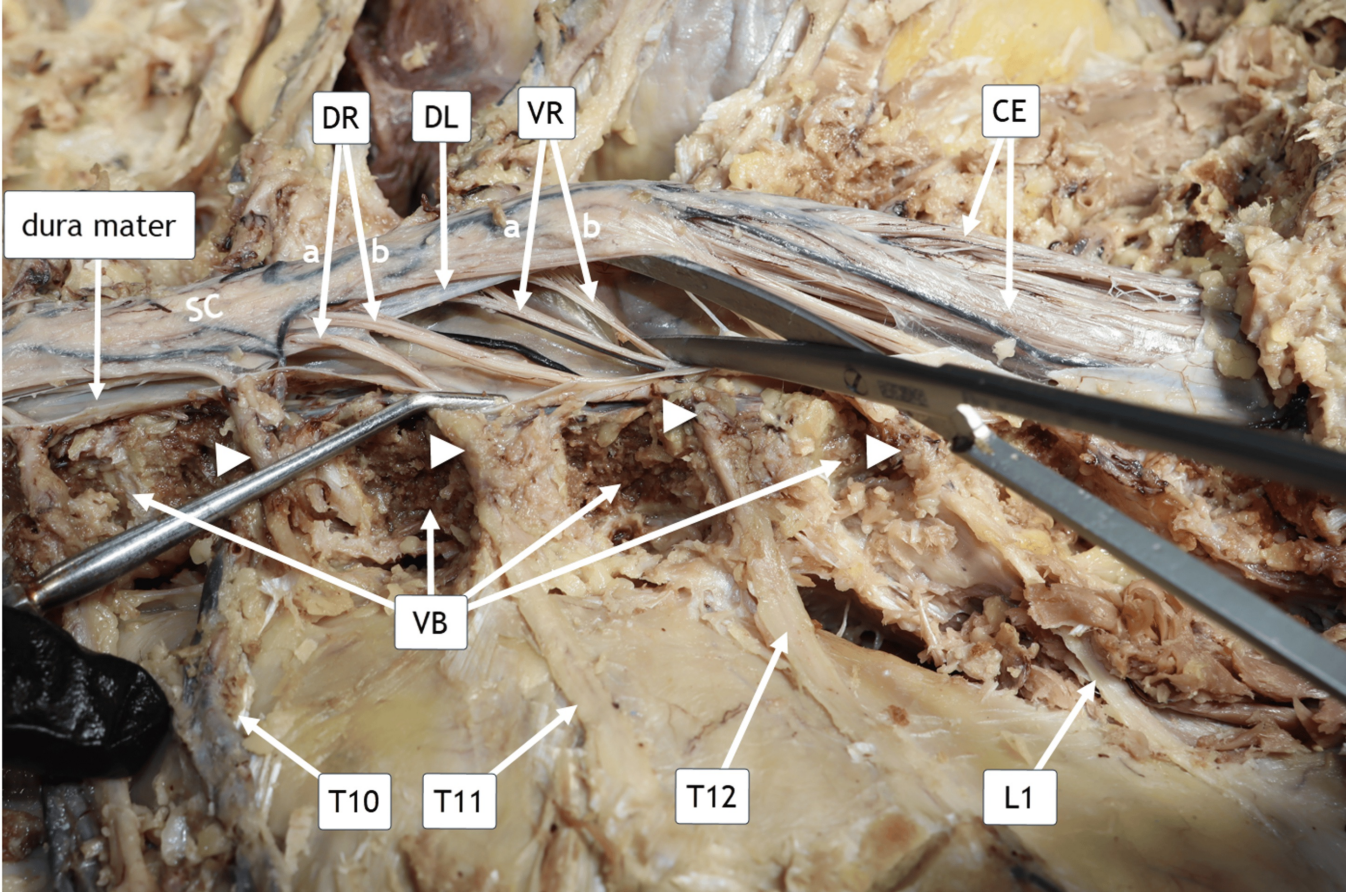
Spinal Cord Rootlets T11 to L1. Horizontal posterior view of the donor in the prone (face-down) position. From the reader’s perspective, the head is oriented toward the left side of the image. The dorsal rootlets, DR(a) and DR(b), correspond to the T11 and T12 spinal nerves, respectively, and course superior to the denticulate ligament (DL) as they enter the spinal cord. The ventral rootlets, VR(a) and VR(b), correspond to the T12 and L1 spinal nerves, respectively, and pass inferior to the DL following their exit from the spinal cord. Arrowheads denote the dorsal root ganglia (DRG), and white arrows identify the cauda equina (CE). Abbreviations: DR(a), dorsal rootlets of T11; DR(b), dorsal rootlets of T12; VR(a), ventral rootlets of T12; VR(b), ventral rootlets of L1; DL, denticulate ligament; VB, vertebral bodies; T10–T12, thoracic spinal nerves 10–12; L1, first lumbar spinal nerve.

The VB from T10 to L1 were carefully carved with chisels to expose regional anatomy. The dura mater was posteriorly incised to reveal the spinal cord. The VB of T10-L1 were further shaved, and the ribs were removed to demonstrate the dorsal root ganglia and their corresponding thoracolumbar spinal nerves. The DR fibers of T11 and T12 are clearly visualized. The VR fibers of T12 and L1, coursing beneath the denticulate ligament (DL), are also identified. The cauda equina (CE) is demonstrated. The *filum terminale* runs centrally in the CE (Figure 8).

## Discussion

This study presents the first complete *in situ* posterior dissection of the human paravertebral sympathetic chain extending continuously from T1 to L1. By preserving the uninterrupted course of the sympathetic trunks and their associated ganglia, rami communicantes, and vertebral relationships, this preparation provides a unique anatomical and educational resource. Unlike previously reported segmented or regionally restricted dissections, the full-length exposure allows direct visualization of longitudinal sympathetic continuity, facilitating a more integrated understanding of autonomic organization and connectivity.

The sympathetic chain ganglia consist of paravertebral collections of efferent neurons that course bilaterally along the vertebral column. These ganglia play a central role in mediating sympathetic autonomic responses, including regulation of cardiovascular tone, heart rate, pupillary dilation, thermoregulation, and visceral function [2]. Owing to their anatomical location, the sympathetic chain and its branches are susceptible to iatrogenic injury during surgical procedures, particularly those employing an anterolateral approach to the cervical spine [12]. This clinical vulnerability underscores the importance of accurate anatomical visualization and understanding of the sympathetic chain’s spatial relationships to surrounding osseous, neural, vascular, and visceral structures. Comprehensive exposure of the intact sympathetic chain therefore enhances both anatomical education and procedural awareness for trainees and clinicians.

Functionally, our findings demonstrate that the sympathetic chain is organized as paired longitudinal trunks positioned bilaterally along the vertebral column, extending across the cervical, thoracic, lumbar, and pelvic regions. Each trunk is composed of sequential paravertebral ganglia interconnected by interganglionic fibers, forming a continuous longitudinal pathway for sympathetic outflow. In our in situ dissections, the sympathetic trunks maintained consistent segmental communication with adjacent spinal nerves through white and gray rami communicantes at their corresponding vertebral levels, reinforcing the integrated relationship between spinal and sympathetic neural pathways. These observations underscore the structural continuity of the sympathetic chain and highlight its role as a coordinated conduit for autonomic signal transmission along the axial skeleton (Figures 5, 6).

As described by Cheng and Tadi, preganglionic sympathetic axons arising from the intermediolateral gray horn of the thoracic and upper lumbar spinal cord enter the sympathetic chain through white rami communicantes, whereas postganglionic fibers exit via gray rami communicantes to rejoin spinal nerves and distribute to peripheral targets. These postganglionic fibers innervate blood vessels, sweat glands, arrector pili muscles, and visceral organs, mediating the physiological responses classically associated with the “fight-or-flight” response [13]. Preservation of these anatomical relationships in the present dissection allows clear visualization of both segmental and longitudinal sympathetic connectivity, reinforcing the functional organization of the thoracolumbar sympathetic system.

Previous investigations of the sympathetic chain have largely relied on anterior or anterolateral approaches and have focused on limited anatomical regions. Street et al. examined anatomical variations of the upper thoracic sympathetic chain in 20 cadavers using an extensive anterior dissection requiring removal of the sternum, clavicles, ribs, scapulae, and thoracic viscera, with analysis restricted to the first four thoracic ganglia [7]. Similarly, Civelek et al. and Saylam et al. employed anterior cervical approaches to visualize cervical sympathetic structures through dissection of neck musculature [5,6]. Additional anterior evisceration-based approaches were reported by Kommuru et al., Chung et al., Zhang et al., and Peetermans et al., focusing primarily on upper thoracic segments or individual rami communicantes [8-11]. While these studies provided valuable region-specific insights, their methodologies precluded preservation of the sympathetic chain within its intact vertebral context and limited appreciation of its full longitudinal organization.

In contrast, the present study employed a posterior dissection approach in a 75-year-old male cadaver. Following systematic removal of posterior musculature, fascia, and adipose tissue, the spinous processes and laminae of the thoracic and upper lumbar vertebrae were carefully removed using an electric saw and chisels. This approach exposed the spinal cord within its dural sac, which was subsequently opened longitudinally to reveal the anterior and posterior rootlets. While anterior approaches avoid extensive bony removal and more closely resemble operative surgical corridors, the posterior approach allowed preservation of the thoracic and abdominal viscera, facilitating uninterrupted visualization of the sympathetic chain within its native vertebral context. This technique also maintained the integrity of surrounding anatomical structures, enabling comprehensive appreciation of sympathetic chain relationships and allowing the specimen to remain suitable for additional anatomical investigations.

Several prior studies, including those by Chung et al., Zhang et al., and Civelek et al., emphasized measurement of ganglionic size and documentation of anatomical variation to inform surgical safety [5,8,9]. While informative, these studies were inherently limited by their restricted anatomical scope. By exposing the sympathetic trunks in their entirety, the present dissection permits observation of continuous interganglionic connections and vertebral-level relationships, offering clinically relevant insights for surgical planning and enhanced anatomical education.

Despite its novelty, this study has limitations. The dissection was performed on a single cadaver, limiting assessment of interindividual anatomical variation. In addition, the presence of a scoliotic curvature introduced asymmetry between the two sides of the dissection, which may affect generalizability and posed technical challenges. The posterior approach was particularly time-intensive, requiring extensive, stepwise reduction of each VB and meticulous removal of the ribs with their associated costovertebral and costotransverse joints to safely visualize the sympathetic chain. The most significant technical challenge involved careful rib and joint removal while avoiding injury to the dorsal root ganglia and preserving the integrity of the sympathetic trunk. A secondary challenge was controlled reduction of the VBs to expose the sympathetic trunk, which lies in close lateral and inferior proximity to each VB. Incremental bone removal was therefore essential to maintain native anatomical relationships and prevent disruption of adjacent neural structures. In total, approximately 260 hours were required to complete this novel dissection.

Future studies should aim to replicate this full-length posterior dissection in larger and more diverse cadaveric cohorts to evaluate variations in ganglionic number, size, and spatial relationships across sex, age, and pathological conditions. Nevertheless, this work establishes a methodological foundation for comprehensive sympathetic chain dissections and provides a valuable reference framework for future anatomical, educational, and clinical investigations.

## Conclusions

This technical report describes a novel and reproducible full-length *in situ* posterior dissection of the human sympathetic chain and its corresponding ganglia from T1 to L1, demonstrated in continuity with the spinal cord from the brainstem to the CE. By preserving ganglia, interganglionic connections, rami communicantes, and native vertebral relationships, this approach overcomes the limitations of previously reported regionally restricted or anterior evisceration-based dissections. The stepwise removal of ribs, costovertebral and costotransverse joints, and controlled reduction of VBs was essential for maintaining neural integrity and anatomical fidelity. This preparation provides a comprehensive anatomical reference that enhances understanding of the thoracolumbar sympathetic system and has important implications for anatomical education, surgical planning, and future investigations of autonomic anatomy *in situ*.
